# Assessment of Occupational Health and Safety among Scavengers in Gaza Strip, Palestine

**DOI:** 10.1155/2020/3780431

**Published:** 2020-02-24

**Authors:** Issam A. Al-Khatib, Majed I. Al-Sari', Stamatia Kontogianni

**Affiliations:** ^1^Institute of Environmental and Water Studies, Birzeit University, Birzeit, State of Palestine; ^2^Universal Institute for Applied and Health Research (UIAHR), The Joint Service Council for Solid Waste Management for Hebron and Bethlehem Governorates (JSC-H&B), Bethlehem, State of Palestine; ^3^Laboratory of Heat Transfer and Environmental Engineering, Dpt. of Mechanical Engineering, Aristotle University of Thessaloniki, Thessaloniki, Greece

## Abstract

This study deals with the occupational health and safety of valuable and recyclable waste collectors (called scavengers) in the Gaza Strip, Palestine. The analytical descriptive approach was used in this study to achieve this goal. Waste pickers in the study area are working informally at existing dumpsites, solid waste transfer stations, landfills, and community streets' bins areas. A sample of 301 scavengers was surveyed filling a structured questionnaire designed for this purpose, during individual interviews. In addition, interviews with key Palestinian officials in the Gaza Strip have been conducted to provide accurate data and comprehensive information regarding waste pickers activities. The results showed that the occupational health and safety of the waste pickers is in constant deterioration mainly due to the informal nature of their work. The waste pickers are reportedly suffering in the current situation and the majority has no access to potable water, sanitation, and hygienically appropriate place to sleep and have meals. None of them has ever received occupational health and safety training. The study recommends that local decision makers should uptake short-term and long-term measures in waste management sector both aiming at improving this vulnerable social group's health and safety life status.

## 1. Introduction

In most cities of the developing countries, thousands of people are depending on the collection of recyclable materials for their livelihoods [1], and it is reported that up to 2% of the population in Asian and Latin American cities lives on scavenging income [2]. Gaza Strip is the largest area under siege in the world. Blockade and restrictions on movement imposed by the Israeli occupation cause deterioration in the local economic conditions. Most of residents are refugees living in deep poverty and unhealthy conditions. The World Bank has reported that the economy is in “free fall” and half the population is living under the poverty line [3]. Mobility restrictions have directly affected the available possibilities for men and women to access health, education, and income, as well as sustain family and other social networks. The economic crisis in the Gaza Strip and lack of access to livelihood has forced several people to work as waste pickers, collecting recyclable materials from the generated municipal solid waste and selling them to manufacturers in order to generate income [4, 5]. The phenomenon of the waste pickers is spread across Palestine in the Gaza Strip as well as in the West Bank. Most of the waste pickers in Palestine are working informally at random dumps. The Wadi Al-Shaer Joint Service Council for Solid Waste Management (WSJSC-SWM) reported the presence of 4 waste pickers at Anabta dumpsite [6]. The Joint Service Council for Solid Waste Management (JSC-H&B) reported the presence of 81 waste pickers were working at Yatta dumpsite, which is located at the southern part of the West Bank, before the closure of the site [7]. Eco Con Serv and Universal Group-Gaza reported the presence of 11 waste pickers at Khan Younis solid waste transfer station and 18 waste pickers at El-Fukhary landfill [8].

In accordance with the interviews held with Joint Service Council for Gaza and North Gaza Governorates, Municipal Development and Lending Fund (MDLF), and the Environmental Quality Authority (EQA), the waste pickers in the Gaza Strip belong into three categories, based on their “job” area:

- Recyclables collection from the bins distributed along the streets in the community
- Informal sorting at the solid waste transfer stations
- Recyclables collection at the dumpsites and landfills

All waste pickers are working informally and independently as they not belonging to any company or organization related to integrated solid waste management. Law prohibits informal work at solid waste management facilities; however, the local authorities in the Gaza Strip are not able to prevent the waste pickers' activity due to sympathy and concerns on their financial status. Despite their occupational unhygienic environment, regulation, monitoring, or even enforcement to regulate their work is not available [9].

Waste pickers belong to poor and marginalized social groups and are often vulnerable to a variety of occupational health risks and diseases [10–13]. They represent the informal grassroots of the local solid waste recycling system, yet they are not taken into consideration by any waste management policy [14–17].

Overall, workers in the solid waste sector including informal waste pickers are vulnerable to three major health risks: accidents, infection, and chronic diseases. Accidents could lead to injury or death and could be caused by heavy equipment, trucks, holding recyclable materials, fire outbreaks, falling from heights when dumping face is high, and buried in the waste. Infections is caused by direct contact with waste and infected wound, infected dust, bites from wild animals, and enteric infections transmitted by insects feeding on waste. Chronic diseases including chronic respiratory diseases result from exposure to dust and toxic and carcinogenic risks as a result of exposure to hazardous compounds, cardiovascular disorders, and heat stress due to exposure to excessive temperature, and hearing function loss result from exposure to excessive noise. Most of the aforementioned incidents frequently occur in most businesses, when training programmes are not regularly implemented.

To this direction, the profession of scavengers is also marked by many risks [18, 19]. Previous studies, worldwide, reveal numerous health problems among the waste pickers population [11, 19–26]. Also, previous studies have shown incidences of infectious diseases among waste pickers as a result of exposure to hazardous substances [27], for example, feces, contaminated needles, toxic paper, heavy metals from batteries, bottles, and chemical waste containers.

A study of 48 waste pickers in Santo André, Brazil, showed that almost all workers reported having back, hand, leg, arm, and shoulder pain [28] due to the lifting of heavy objects.

In particular, waste pickers working in open dumps are exposed to large quantities of toxic vapors and other serious threats such as the possibility of being run over by dump trucks to landfill, fire exposure, or falling due to surface decline. They are exposed to nonfatal and sometimes fatal occupational hazards and accidents. However, some studies have showed increased risk of musculoskeletal problems [29] and work-related respiratory gastrointestinal and skin problems [30]. The exposure to microbes as well as dust during their work is likely to have cumulative effect and health problems to emerge in the long term [31]. Usage of protective equipment (gloves and uniform) may, in large extent, minimize the involved risks. Yet, studies have revealed very low usage rates [21, 22, 32]; hence, the scavengers are vulnerable to injuries by sharp objects (needles, broken glass, metal, and so on) and animals (dog bites, rat bites, and so on) [33].

Municipal cleaners also face similar problems as scavengers based to a study conducted in the Nablus governorate [34]. This has been also crosschecked with the Environmental and Social Impact Assessment outcome of Al-Minya sanitary landfill in the southern West Bank of Palestine [35].

The issues described so far do not constitute common knowledge in Palestine and in the Gaza Strip; therefore, the aim of this study is to identify in the field and communicate the occupational hazards that scavengers are exposed to in the Gaza Strip.

## 2. Research Methodology

The study population consists of all scavengers working in the Gaza Strip. A random sample of the study population was selected in accordance with the Herbert Larkin equation [36], as shown in the following equation.

(1) $$\begin{array}{c} n=\frac{\left(1-p\right)}{\left(\text{SE}|/|t\right)+\left[p\left(1-p\right)|/|N\right]}, \end{array}$$

where *N* = study population (1200); *n*: sample size; *t*: confidence coefficient and equal to 1.96 for 95% confidence interval; *p* is the value of the main estimate, which is a relative index assumed to be 50% in order to give the largest sample size possible for this type of indicator; SE: the standard error ratio is equal to 0.05. A minimum sample size of 291 is needed. A sample of 301 scavengers was interviewed.

The data were collected via a structured questionnaire designed for the purpose that is to record comprehensive information regarding occupational health and safety of the waste pickers. Questionnaires were provided during personal interviews with the waste pickers. Prior to the research conduction, the questionnaire was tested in pilot-interviews, and appropriate modifications were performed. It included the following sections: general information, awareness and education, occupational health and safety, social aspects, and trade unions. The overall focus was on the workplace environment status, working hours, accidents recording, perceptions of occupational hazards, recorded occupational diseases, availability of safety equipment, customized safety procedures, the culture and awareness, availability of health insurance, provided medical services, and other related topics. All participants were informed of the field research aims prior to the interview conduction, to facilitate the data collection process and to obtain their prior consent to data collection.

Another part of the overall study was the communication with relevant decision-makers and specialists of the Palestinian officials related to the research scope, namely, the Joint Service Council for Gaza and North Gaza Governorates, the Municipal Development and Lending Fund (MDLF), and the Environmental Quality Authority (EQA). This communication largely assisted the cross reference of the interviews' collected data and its connection to current situation.

## 3. Results and Discussions

### 3.1. Socioeconomic Conditions of the Waste Pickers (Scavengers)

The study sample was selected to represent all parts of the Gaza Strip. The distribution of the sample was 20.6% from the north Gaza district, 26.9% from Gaza district, 10.6% from Deir Al-Balah area, 33.6% from Khan Younis, and 8.3% from Rafah area. The results showed that all of the waste pickers are males. The socioeconomic status of the study sample is shown in Table 1.

The socioeconomic conditions analysis showed that 50.2% are aged between 19–30 years; 52.5% of them are married; 83.4% are permanent city residents; and 62.5% belong to families consisting of 5–10 members. The vast majority of the waste pickers are living in deep poverty; the average monthly income generated from working in the waste recycling is less than NIS 500 for 71.9% of them (1 USD ⋍ 3.5 NIS), and the education level of 60.5% of them is that of preparatory or secondary school. In comparison with other studies, Schenck et al. [37] found that 47% of the waste pickers are married in Pretoria. In accordance with Figueiredo et al. [38]; 56% of the waste pickers are aged between 18–34, 72% of them were of primary education level, and 72% of them are generating income in the range of 300–500 BRL (1 BRL ⋍ 0.25 USD) from working in collection and selling recyclables. Schenck et al. [39] found that the average age of the waste pickers was 39 years. According to Women in Informal Employment, Globalizing and Organizing [40], 43% of 760 surveyed waste pickers were in the age range of 26–40 years. Moreover, 49% of the waste pickers were aged in the range of 41–50 years according to Schenck et al. [37]. The United Nations Inter-Agency Task Force on Social and Solidarity Economy reported that International Labor Organization (ILO) has estimated approximately 15–20 million informal waste workers worldwide generating a very low income, often living below the poverty level [41]. In 2012, Schenck et al. conducted a study on waste pickers' population in South Africa and found out that the percentage of men was slightly higher (52%) than the percentage of women (48%). Schenck et al. [39] found that 60% of the waste pickers were men and 40% were women.

The working hours for most of the waste pickers range between five and twelve hours per day; five to eight for 55.3% and nine to twelfth for 39.7% of them. In the West Bank/Palestine, *the Joint Service Council for Solid Waste Management for Hebron and Bethlehem Governorates -JSC-H&B* [7] reported that the waste pickers were working 8 hours daily at Yatta dumpsite during the period when waste was still disposed there. Asim et al. [42] noted that the waste pickers were working an average of 10 hours per day in Pakistan. Thirarattanasunthon et al. [43] showed that waste pickers were working between 6 and 18 hours per day in Thailand.

### 3.2. Health Insurance, Professional Union, and Responsible Body

99.0% of waste pickers reported that their safety is not guarded by any government agency, and 87.0% of them are not belonging to any professional union. However, 90.4% of them have health insurance, where 22.3% is governmental health insurance, 39.1% health insurance offered by the United Nations Relief and Works Agency for Palestinians in the near east (UNRWA) as most of the residents of the Gaza Strip are refugees, and 35.4% have both governmental and UNRWA health insurance. Interviews with key Palestinian environmental officials (*Joint Service Council for Gaza and North Gaza Governorates*, *Municipal Development and Lending Fund (MDLF)*, *and the Environmental Quality Authority (EQA))*, confirmed that all waste pickers are working informally for themselves with no responsible body for their safety. In north Gaza area, the number of waste pickers has been identified and in order to minimize the risks and the number accidents, the responsible personnel issued a particular time frame they were allowed to work in the landfill cells. In addition, the *Joint Service Council for Solid Waste Management, North Gaza Branch,* decided to restrict the access to the landfill to all (to avoid accidents). Any waste picker enters on his own responsibility having signed an obligation that releases the responsibility of the *Joint Service Council for Solid Waste Management* toward the waste picker.

### 3.3. Occupational Health Diseases

**Figure 1:**
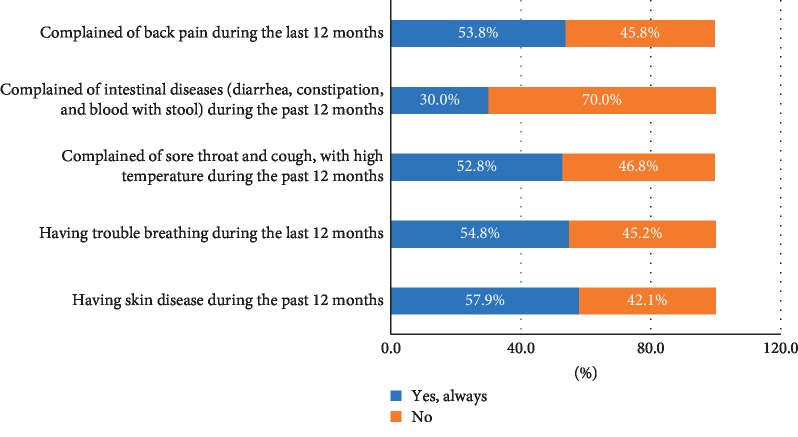
Occupational diseases of the waste pickers.

The waste pickers were requested to describe any health problem they faced during the last 12 months. More than 50% of the study sample answered that they were troubled by back pains, breathing issues, skin diseases, sore throat, and cough with high temperature. However, only 30% complained of intestinal diseases (diarrhea, constipation, and blood with stool), as shown in Figure 1.

Gogoi [44] reported the common diseases affecting scavengers, namely eye irritation (88%), asthma (76%), cold and cough (92%), fatigue (94%), stomach problems (20%), and back pain (96%). Leton and Nweke [45] studied the health risks of scavengers in 15 dumpsites in Nigeria and found that pending positions and carrying heavy loads of recyclable materials cause backache, pain in legs and arms, and stiffness of joints. Thirarattanasunthon et al. [43] reported that most of the waste pickers suffered from sprains and pains in the lower back (95%), skin rashes (66%), common colds (89%), fatigue (34%), headaches (49%), and shortness of breath (23%). Diarrhea incidence among informal waste pickers was found to be 10 times greater compared to the general population data [46].

In addition, the surveyed waste pickers were investigated as per their immunization against viral hepatitis and tetanus. The results showed that 61.1% of them received vaccination against viral hepatitis and 66.4% received vaccination against tetanus. Vaccination against infectious disease, including tetanus, hepatitis A, and hepatitis B is a successful protective measure that significantly reduces the risks associated with contaminated materials contact [47]. Limited source-separation practices in the municipal waste stream in Palestine means that certain hazardous fractions such as sharp objects, batteries, and medical wastes exist in the waste stream, increasing the vulnerability to risks and the cases of occupational-related diseases. Hunt [48]; highlighted that the official formalization of the waste sorting and recycling activities that can contribute to the protection the workers from health hazards, are at the same time measures for hazardous waste sustainable disposal. To this direction, the risks associated with the collection process can be managed together with the risks of individuals involved in the official collection processes or unofficial one (waste pickers).

### 3.4. Occupational Safety

The waste pickers were requested to identify safety issues that troubled them during that last 12 months. The results showed that to 58.3% of them burn injury occurred, and 90.4% have been exposed to hazardous waste materials such as neglected medicine, bandages, dead animals, paints, batteries, and sharp objects like shaving blades. However, more than three-fourth of them had no incident of sprained foot or joint, a joint dislocation or fracture, and tooth fracture during the last 12 months, while 53.3% of them have never experienced any muscle tear. The results are shown in Table 2.

Parizeau [49] reported exposure of the waste pickers to traffic accidents, broken bones, cuts from glass and sharp metals found in the trash, tiredness and fatigue, burns, aches and pains, and breathing problems. Nguyen et al. [32] also reported that scavengers suffer from pains in the back, headaches, coughs, sore muscles, stomach pains, and rashes, and nearly all respondents of his study suffered from cuts in hands, limbs, and feet. In a comparative study conducted by da Silva et al. [50] between informal waste pickers group and controlled one of similar socioeconomic conditions found that the incident of back pain was similar in both groups pointing the finger to hard work without proper equipment.

### 3.5. Use of Safety Tools and Hygiene Practices

**Figure 2:**
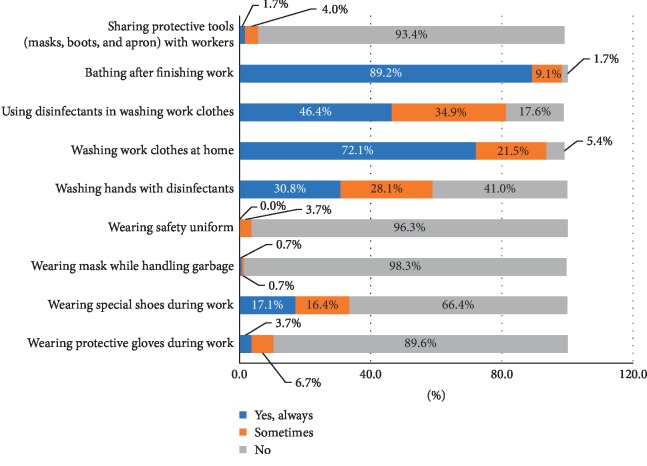
Use of safety tools and hygiene practices of the waste pickers.

Scavengers are facing risks similar to the workers in the waste sector, yet in a severe level beyond the expected common diseases such as wounds, cuts in different parts of the body, toxic animal bites, burns and inhalation difficulties due to exposure to chemicals and toxic gases, traffic accidents and falls, and musculoskeletal problems, which are all part of a waste pickers' daily burden [51]. The use of safety tools, personal protective equipment (PPE) and good hygiene practices can, to a large extent, reduce vulnerability to occupational risks. The International Labor organization (ILO) recommendations measures to improve safety of waste pickers which include the use of gloves, safety shoes, tools for waste sorting, and vaccination against tetanus [52]. Limited awareness on potentially risky materials handling together with the limited use of protective equipment makes even household waste hazardous and poses health risks to handlers [47]. The research results showed that more than two-thirds of the waste pickers only *sometimes* make use of the protective equipment by sharing it; uniforms, masks, hard-covered shoes, and protective gloves, during working hours. It is rather alarming that protective equipment is shared among them, since it is a serious reason for the enhancement of diseases transmission among scavengers. However, 72.1% of them are always washing their clothes at home, 89.2% are always bathing after finishing their work, 46.4% are always using disinfectant in washing work clothes, and only 30.8% are always washing their hands using disinfectants. The results are shown in Figure 2.

Those are considered as good practices that secure the scavengers' population health level and enhance protection. Nyathi et al. [53] found that only 27.4% of women scavengers did not use self-protection attire and 69.2% of men scavenger never used self-protection attire in South Africa. He also found that washing and cleaning of the body is immediately performed by female scavengers after the day's work in the temporary shacks using soap, while men do not clean themselves at work place but do so once they return back home.

**Figure 3:**
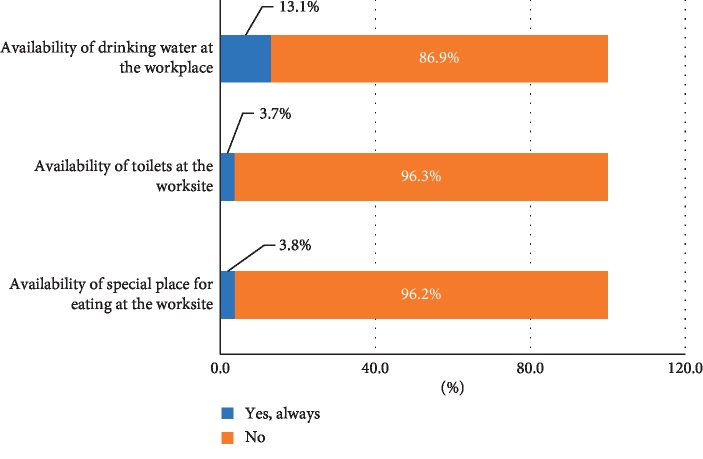
Access to water and sanitation services at the worksite.

In addition, access to clean water and sanitation services, as well as availability of hygienic-appropriate places for meals is another yet important issue that enhances occupational health protection level. The vast majority of the waste pickers reported that clean drinking water is not available at the workplace (86.9%), neither toilets (96.3%), nor an appropriate meal place at worksite, as shown in Figure 3. Additionally, the aforementioned basic needs are not covered neither at their homes, undercutting the overall hygienic quality of the population.

### 3.6. Health and Safety Training

Occupational health and safety training in the field is essential, and it is ascertained that it contributes to the reduction of work accidents and occupational diseases. Nearly no respondents receive any training related to the nature of the work they are carrying out (99.7%). However, the International Labor organization (ILO) has suggested the implementation of health and safety training seminars, regular health check-ups, and monitoring of waste pickers in order to safeguard them [52]. Baker et al. [54] conducted a study on the empowerment of informal waste pickers and their active participation to the official municipal solid waste management sector; he found out that waste pickers are highly capable, after a limited training period, to acquire entrepreneurial skills and highly contribute to sustainable solid waste management. The regulation of their work undoubtedly leads to local circular economy and life status enhancement together with the risks exposure reduction.

## 4. Conclusions and Recommendations

Given that the information on waste pickers' livelihood is limited in Palestine, in the Gaza Strip in particular, and this study aims to identify their current occupational health and safety status. Data collection via structured questionnaires and direct interviews by 301 waste pickers were backed up with comprehensive information provided by the communication with local key environmental experts.

The results showed that all of the waste pickers in the Gaza Strip are self-employed and are not belonging to any official company or agency. The vast majority of them have health insurance policy issued even by the government or by the UNRWA.

Occupational health and safety level of the waste pickers are limited since during the last 12 months; more than 50% of the waste pickers suffered from occupational-related diseases, (30%) from intestinal diseases. On that note, 65% have burns; the majority has been exposed to hazardous materials. Nevertheless, more than 75% have not experienced any physical incident (sprained foot, sprained joint, dislocation, fracture, and tooth fracture) during the last 12 months and around 65% of them have been vaccinated against hepatitis and tetanus.

Overall, more than two-thirds of the waste pickers are using protective equipment (even on a shared basis) and are washing their clothes, but less than half of them use disinfectants regularly. Most importantly, the majority of waste pickers have no access to potable water, sanitation units, and hygienically appropriate place for having meals neither at workplace not at home, which is rather alarming for their health status level. Almost none of the waste pickers have received any occupational health and safety training.

It has been studied and reported that waste pickers' activity may enhance local waste management sector and lead to landfilled solid waste reduction. Solid waste management officials are encouraged to invest in occupational training of the scavengers' population to enhance their skills and provide them with the opportunity to enhance their living status in the meantime by minimizing the waste-borne hazards and vulnerabilities [55, 56]. That way, in the process of waste separation improvement, waste pickers will simultaneously improve the current occupational health and safety level and gain access to potable water and sanitation. Their formalization attempt may be implemented in collaboration with associations or local companies where safeguarding polices are applicable. Public/private partnership between local authorities and waste pickers associations can eliminate informal recyclables collection and reduce, largely, vulnerability of the waste pickers to risks.

## Figures and Tables

**Table 1:** Socioeconomic conditions of the waste pickers in the study area.

Variable	Results					
Age	0–9	10–18	19–30	31–50	≥50	Total
	1.0%	24.0%	50.2%	21.3%	3.3%	(100%)
Marital status	Single	Married				Total
	47.2%	52.5%				100.0%
Permanent residence	City	Refugee Camp	City			Total
	83.4%	10.6%	6.0%			100.0%
Number of family members	≤4	5–10	11–15	16–20	>20	Total
	20.9%	62.5%	10.6%	1.3%	0.7%	100.0%
Average monthly income (NIS) from working in waste collection	<500	500–1000	>1000			Total
	71.9%	27.2%	0.7%)			100.0%
Level of education	Illiterate	Elementary	Preparatory and secondary	College or university		Total
	5.0%	30.6%	60.5%	4.0%		100.0%
Daily working hours	≤4	5–8	9–12	>12		Total
	3.7%	55.3%	39.7%	1.3%		100.0%

**Table 2:** Occupational safety of the waste pickers.

Variable	Answer		
	Yes, always	No	Total
Having sprained foot during the last 12 months	61 (20.3%)	238 (79.3%)	300 (100%)
Having a sprain in the joint during the last 12 months	47 (15.6%)	254 (84.4%)	301 (100%)
Having joint dislocation during the last 12 months	37 (12.3%)	263 (87.7%)	300 (100%)
Having muscle tear during the last 12 months	140 (46.7%)	160 (53.3%)	300 (100%)
Having tooth fracture in the last 12 months	53 (17.6%)	248 (82.4%)	301 (100%)
Having scratches/injuries during the last 12 months	293 (97.7%)	7 (2.3%)	300 (100%)
Having fracture during the last 12 months	60 (20.1%)	239 (79.9%)	299 (100%)
Having burns during the last 12 months	175 (58.3%)	125 (41.7%)	300 (100%)
Exposure to hazards (such as neglected medicines, bandages, dead animals, paints and batteries, and sharp objects such as shaving blades, glass, or metal parts) during the last 12 months	272 (90.4%)	29 (9.6%)	301 (100%)

## Data Availability

The data used to support the findings of this study are available from the corresponding author upon request.
